# Within-Host Bacterial Diversity Hinders Accurate Reconstruction of Transmission Networks from Genomic Distance Data

**DOI:** 10.1371/journal.pcbi.1003549

**Published:** 2014-03-27

**Authors:** Colin J. Worby, Marc Lipsitch, William P. Hanage

**Affiliations:** Center for Communicable Disease Dynamics, Department of Epidemiology, Harvard School of Public Health, Boston, Massachusetts, United States of America; Duke University, United States of America

## Abstract

The prospect of using whole genome sequence data to investigate bacterial disease outbreaks has been keenly anticipated in many quarters, and the large-scale collection and sequencing of isolates from cases is becoming increasingly feasible. While sequence data can provide many important insights into disease spread and pathogen adaptation, it remains unclear how successfully they may be used to estimate individual routes of transmission. Several studies have attempted to reconstruct transmission routes using genomic data; however, these have typically relied upon restrictive assumptions, such as a shared topology of the phylogenetic tree and a lack of within-host diversity. In this study, we investigated the potential for bacterial genomic data to inform transmission network reconstruction. We used simulation models to investigate the origins, persistence and onward transmission of genetic diversity, and examined the impact of such diversity on our estimation of the epidemiological relationship between carriers. We used a flexible distance-based metric to provide a weighted transmission network, and used receiver-operating characteristic (ROC) curves and network entropy to assess the accuracy and uncertainty of the inferred structure. Our results suggest that sequencing a single isolate from each case is inadequate in the presence of within-host diversity, and is likely to result in misleading interpretations of transmission dynamics – under many plausible conditions, this may be little better than selecting transmission links at random. Sampling more frequently improves accuracy, but much uncertainty remains, even if all genotypes are observed. While it is possible to discriminate between clusters of carriers, individual transmission routes cannot be resolved by sequence data alone. Our study demonstrates that bacterial genomic distance data alone provide only limited information on person-to-person transmission dynamics.

## Author Summary

With the advent of affordable large-scale genome sequencing for bacterial pathogens, there is much interest in using such data to identify who infected whom in a disease outbreak. Many methods exist to reconstruct the phylogeny of sampled bacteria, but the resulting tree does not necessarily share the same structure as the transmission tree linking infected persons. We explored the potential of sampled genomic data to inform the transmission tree, measuring the accuracy and precision of estimated networks based on simulated data. We demonstrated that failing to account for within-host diversity can lead to poor network reconstructions - even with repeated sampling of each carrier, there is still much uncertainty in the estimated structure. While it may be possible to identify clusters of potential sources, identifying individual transmission links is not possible using bacterial sequence data alone. This work highlights potential limitations of genomic data to investigate transmission dynamics, lending support to methods unifying all available data sources.

## Introduction

Population genomic studies have become essential tools in studying the global spread [1] and evolutionary adaptation [2] of infectious agents. Falling costs and technological advances offer the prospect of using whole pathogen genome sequences to investigate individual, localized outbreaks and identify chains of transmission. The ability to identify infection routes would contribute much to the understanding of transmission dynamics, contact patterns in an at-risk population and the optimization of infection control strategies [3]. However, until now, attempts to estimate transmission networks have relied on sparse data and simplifying assumptions, including genetically homogeneous carriage and/or transmission [4], [5], [6], [7], [8]. Within-host populations of bacterial pathogens may be heterogeneous, as recent studies have begun to show, and in such cases characterizing an infection by a single isolate may be misleading. Aspects of bacterial carriage and transmission are still poorly understood, making the interpretation of genomic data collected from outbreaks far from straightforward. As whole genome sequence data for bacterial pathogens become ever more abundant, it is important to understand both the potential and limitations associated with this information.

### Bacterial genetic diversity within and between host

A bacterial population of size 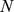, which is initially genetically homogeneous, diversifies over time due to the random introduction of mutations at rate 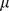 per genome per generation. While there are many measures of diversity, we consider the expected pairwise genetic distance (eg. number of single nucleotide polymorphisms (SNPs)) observed when sampling two random isolates from the population;
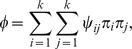
where 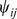 is the genetic distance between variants 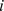 and 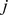, whose respective frequencies are 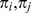.

Under neutral assumptions, the expected pairwise SNP distance at equilibrium is 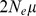 [9], where 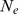 is the effective population size, and 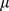 is the mutation rate. However, equilibrium dynamics cannot typically be assumed for within-host carriage of a bacterial pathogen. An initially clonal population takes a considerable amount of time to reach equilibrium levels of diversity (Figure S1).

Evidence has recently emerged that in some pathogens within-host genetic diversity is common. In principle, an individual may harbor a diverse pathogen population due to one or more of the following: infection with a diverse inoculum, diversification of the population due to mutation or other genetic change during infection, and multiple infections from different sources. Studies of *Staphylococcus aureus* have revealed carriage of multiple sequence types, likely caused by independent transmission events [10], [11], as well as diversification over time in long-term carriers [12], [13], and the coexistence of several genotypes, differing by several SNPs [14], [15]. *Streptococcus pneumoniae* populations in an individual may harbor genetically divergent lineages, as has long been appreciated [16]. Within-host diversity of other bacterial pathogens has been studied less frequently, although there is some evidence for heterogeneous carriage of *Helicobacter pylori* [17], *Pseudomonas aeruginosa* [18], *Burkholderia dolosa* [19] and *Klebsiella pneumoniae* [20].

A transmission event involves passing a sample (inoculum) of bacteria from a carrier to a susceptible individual. This is an example of a population bottleneck, as a small fraction of the original population is allowed to independently grow and mutate in a new environment. Assuming the inoculum is a random sample of size greater than 1, it can be shown that the expected sample diversity is equal to that of the original population regardless of the size of the bottleneck (see *Supporting Information*). However, the variance of the expected diversity is inversely proportional to the size of the bottleneck (Figure S2), demonstrating that small bottlenecks may generate considerably different levels of diversity in the recipient due to stochastic effects. Estimating the bottleneck size associated with transmission is challenging, not least because estimates of pathogen diversity pre- and post-bottleneck will be based on a finite sample, and will themselves be uncertain. A wide bottleneck has previously been implicated in the transmission of equine [21] and avian [22] influenza, while inoculum size for bacterial pathogens may vary dramatically [23].

### Inference of transmission routes

There have been several studies aiming to reconstruct transmission links using genetic data. Many have relied on a phylogenetic reconstruction of available isolates, under the assumption that the transmission network will be topologically similar to the estimated phylogeny [5], [8], [24], [25], [26]. However, the phylogenetic tree will not generally correspond to the transmission network based on samples collected during an outbreak [27], [28], [29]. Furthermore, within-host diversity and heterogeneous transmission – the transmission of a genetically heterogeneous inoculum to a new host – will typically complicate such an approach, as isolates from one individual may potentially be interspersed within the same clade as those from other carriers. Under certain assumptions, the molecular clock can be used to dictate the plausibility of a transmission event. As the estimated time to the most recent common ancestor (TMRCA) between isolates sampled from two carriers gets further from the estimated time of infection, the probability of direct transmission falls, and a cutoff can be specified, beyond which transmission is deemed impossible (eg. [30]). This approach requires homogeneous transmission and a robust estimate of the mutation rate. Other network reconstruction approaches have used weighted graph optimization [4], as well as Markov chain Monte Carlo (MCMC) algorithms to sample over all possible transmission links [6], [7].

Several variables may affect the outcome of such analyses. Firstly, the method and frequency of sampling is of great importance. Taking one sample per case ignores within-host diversity and could lead to poor estimates of the genetic distance between cases. Asymptomatic infections may not be detected, or may only be detected long after the time of infection – this can lead to greater uncertainty in the estimated network. Secondly, the bottleneck size plays a crucial role in the amount of diversity established in the newly infected host. Thirdly, the infectious period affects the degree of diversity that may accumulate within-host, and therefore gets passed on to susceptible individuals.

Using phylogenetic reconstruction as a means to estimate transmission is often inappropriate [29], and even when combined with additional analytical methods designed to infer transmission, produces highly uncertain networks [31]. Furthermore, such methodology typically cannot account for diverse founding populations. We instead used a genetic distance-based approach to determine how informative genomic data can be when used to estimate routes of transmission. Many methods aiming to reconstruct either phylogenetic trees or transmission networks are based on a function of a pairwise genetic distance matrix. These include graph optimization [4], the MCMC sampling approaches [6], [7], and various tree reconstruction methods (eg. neighbor joining, unweighted pair group method with arithmetic mean (UPGMA), minimum spanning tree). As such, we used a generalized weighting function based on genetic distance to reconstruct networks, in order to provide a framework flexible enough to be similar (or, in some cases, equivalent) to these methods. We investigated how accurately transmission networks could be recovered, and how accuracy was affected by factors such as bottleneck size, transmission rate and mutation rate. We simulated disease outbreaks under a variety of scenarios, and reflecting various sampling strategies. Our approach could accommodate within-host diversity and variable bottleneck sizes, in order to investigate their effect on network reconstruction. Full details are given in *Materials and Methods*.

## Results

### Within-host diversity

**Figure 1:**
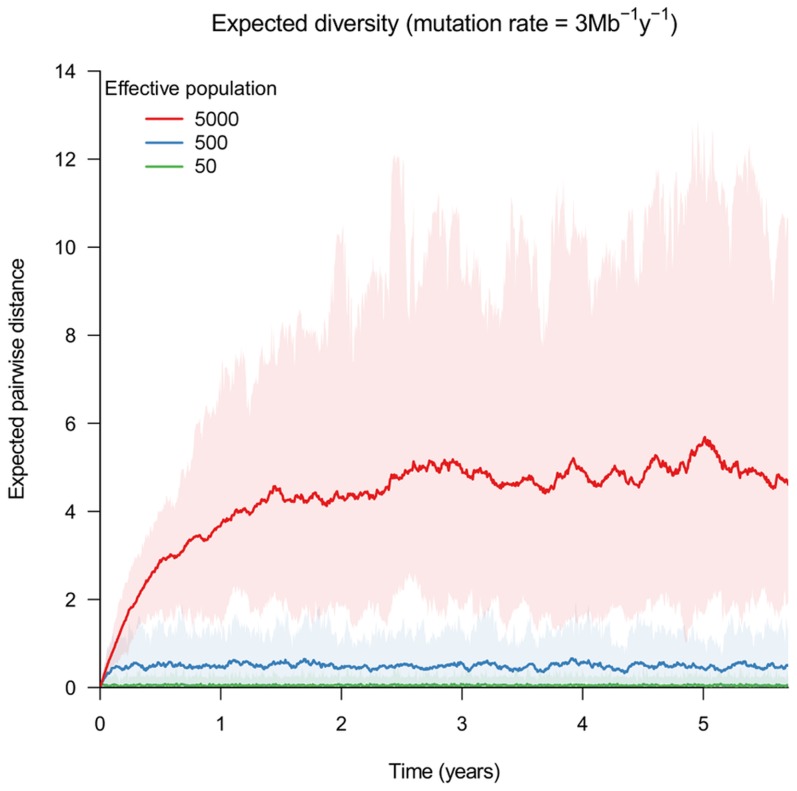
The development of diversity from an initial clonal population, using parameter estimates for *S. aureus*. The generation time was 30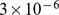 per site per year, and we used an effective population size in the range of 50–5000.

We first simulated diversification within a single host, using *S. aureus* as an example, and compared our findings with estimates of diversity based on published samples. The expected genetic pairwise distance for *S. aureus* carriage has been estimated at 4.12 SNPs [15]. *S. aureus* has a mutation rate of approximately 5×10^−4^ per genome per bacterial generation (given a rate of 3×10^−6^ per nucleotide per year [1], [12] and a generation time of 30 minutes [32], [33], [34]). Nasal carriage of *S. aureus* has been estimated to have an effective population size in the range 50–4000 [12], [15]. Figure 1 shows the accumulation of diversity over time under these parameters. Our simulations indicate that if we assume a host acquires a homogeneous transmission, the expected colonization period required for previously observed levels of diversity to emerge under neutral evolution is typically long (∼1 year). While *S. aureus* may be carried for a number of years [35], observing high diversity from recently infected individuals suggests that alternative explanations may be more realistic. First, repeated exposure to infection may result in the introduction of new strains to a host, potentially resulting in rapid establishment of diversity. Second, the transmitted inoculum may not be a single genotype, but rather a sample of genotypes from the source. This was investigated in detail in the next simulation experiments.

### Transmission chains

We assessed the effect of bottleneck size in a disease outbreak by firstly considering a simple transmission chain, where each infected individual transmits to exactly one susceptible individual. We considered an initial bacterial population of 10 genotypes, which had an expected pairwise distance of 5 SNPs, which could represent a long-term carrier, or the recipient of a diverse infection. We then simulated a transmission event by selecting an inoculum of size 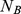. We allowed the new founding population to reach equilibrium population size and imposed another bottleneck after 1000 generations. We repeated this process for 25 transmission events. Figure S3 shows six realizations of our simulations under different values of 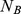. Clearly, while diversity rapidly drops away for small bottlenecks, larger sizes (>10 cells) allow diversity to persist for several bottlenecks. With sufficient mutation between transmission events, diversity can be maintained (Figure S4).

If bacterial specimens taken from disease carriers in an outbreak are sequenced, we can attempt to estimate the routes of transmission based on the genetic similarity of the isolates. There are a number of additional factors that may inform our estimate of the transmission route, such as location, contact patterns and exposure time, but we examined the information to be gained from sequence data alone. More than one isolate may be taken from a carrier, sampled either simultaneously or at various time points during infection, necessitating a choice of how to describe the genetic distance between *populations* of isolates from two cases. We considered both the mean pairwise distance and the centroid distance to summarize the genetic distance between groups of isolates, but found that both resulted in very similar network reconstructions. Network edges were given a weighting which we assume is inversely proportional to the genetic distance (see *Materials and Methods* for detailed specification of weighting functions).

**Figure 2:**
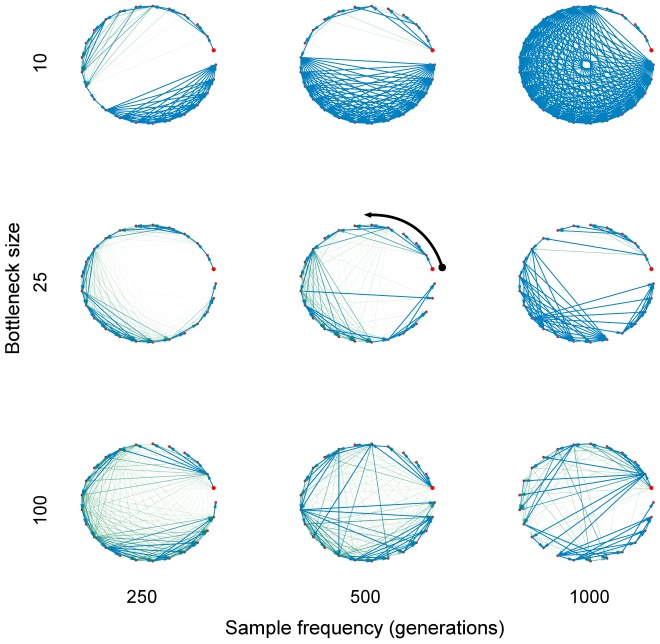
Estimated transmission networks, based on periodical sampling of isolates. The true transmission chain begins with individual identified by the large red dot, and proceeds around the circle as directed by the black arrow. The first individual had a heterogeneous infection, with an expected pairwise distance of 5 SNPs. Each network represents a single estimate of a simulation, with edge weighting proportional to the relative probability of infectious contact, inversely proportional to the mean genetic distance between individuals. It was assumed that the order of infection is known, such that the 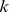
th infection has 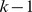 potential sources.

**Figure 3:**
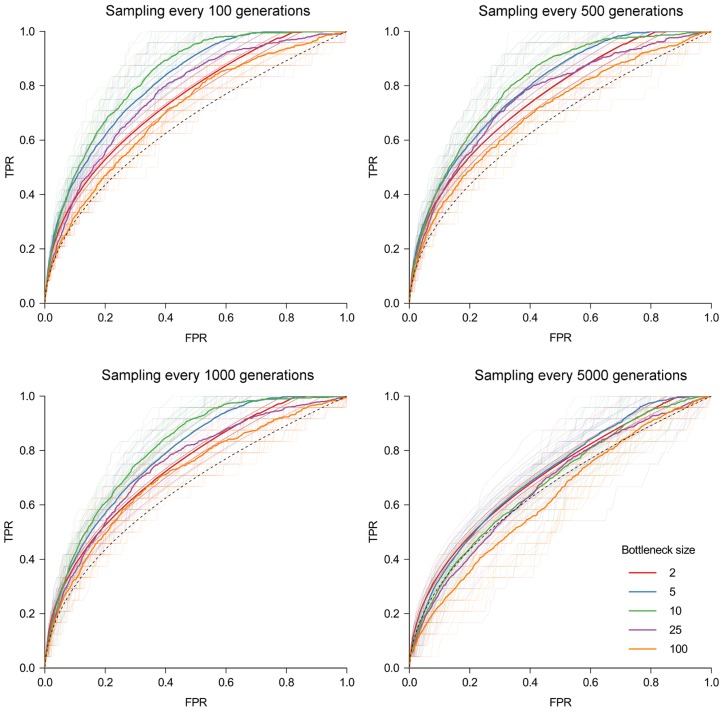
ROC curves for estimated networks under various scenarios. Under each scenario, 25 datasets were simulated. Lighter lines indicate the ROC curve for a particular replication, while the heavier lines indicate the mean ROC curve for a given scenario. The dashed line indicates the ‘no information’ ROC curve, where sources are guessed at random. It was assumed that the order of infection is known, such that individual 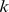 has 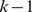 potential sources (meaning that guessing sources at random produces an ROC curve above the diagonal). TPR: true positive rate; FPR: false positive rate.

The single transmission chain provides an idealized scenario to reconstruct transmission links. Furthermore, we assumed that the order of infection is known. As such, the potential source for each individual 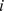 can only be one of the preceding 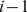 generations, which, intuitively at least, should become more genetically distant as one goes farther back in time. Transmission events occur every 1000 bacterial generations, and one cell is selected randomly from each individual's bacterial carriage at regular intervals (possibly more frequent than the transmission process) for sequencing. Figure 2 shows reconstructed networks for a range of scenarios. We repeated this for several simulations under each scenario, and plotted receiver-operating characteristic (ROC) curves to assess the accuracy of the reconstructed network (Figure 3). We observed that there was an optimal bottleneck size in this setting which allows the network to be resolved with a relatively high level of accuracy; for the scenario considered here, networks reconstructed using a bottleneck size of 10 clearly outperform those constructed using both larger and smaller inoculum sizes. In this setting, larger bottlenecks allow a very similar bacterial population to be established within each new infective, while smaller bottlenecks rapidly result in a single dominant strain being carried and transmitted by the infected population. The optimal bottleneck size depends on the outbreak size, as well as the expected change in pathogen diversity within-host between time of infection and onward transmission. We found that infrequent sampling (eg. one sample per infected individual) can lead to a reconstruction that is no better than selecting sources at random, and sometimes worse.

### Epidemics

We next considered a more general susceptible-infectious-removed (SIR) epidemic, in order to determine how network accuracy is affected by transmission and mutation rate, and sampling strategy. We again estimated the transmission network based upon observed sequence data alone under the assumption that the order of infection was known. Both the centroid and pairwise distance metrics were used, but we found that the performance of both was very similar. For this reason, all results shown here have been derived using the pairwise distance measure.

**Figure 4:**
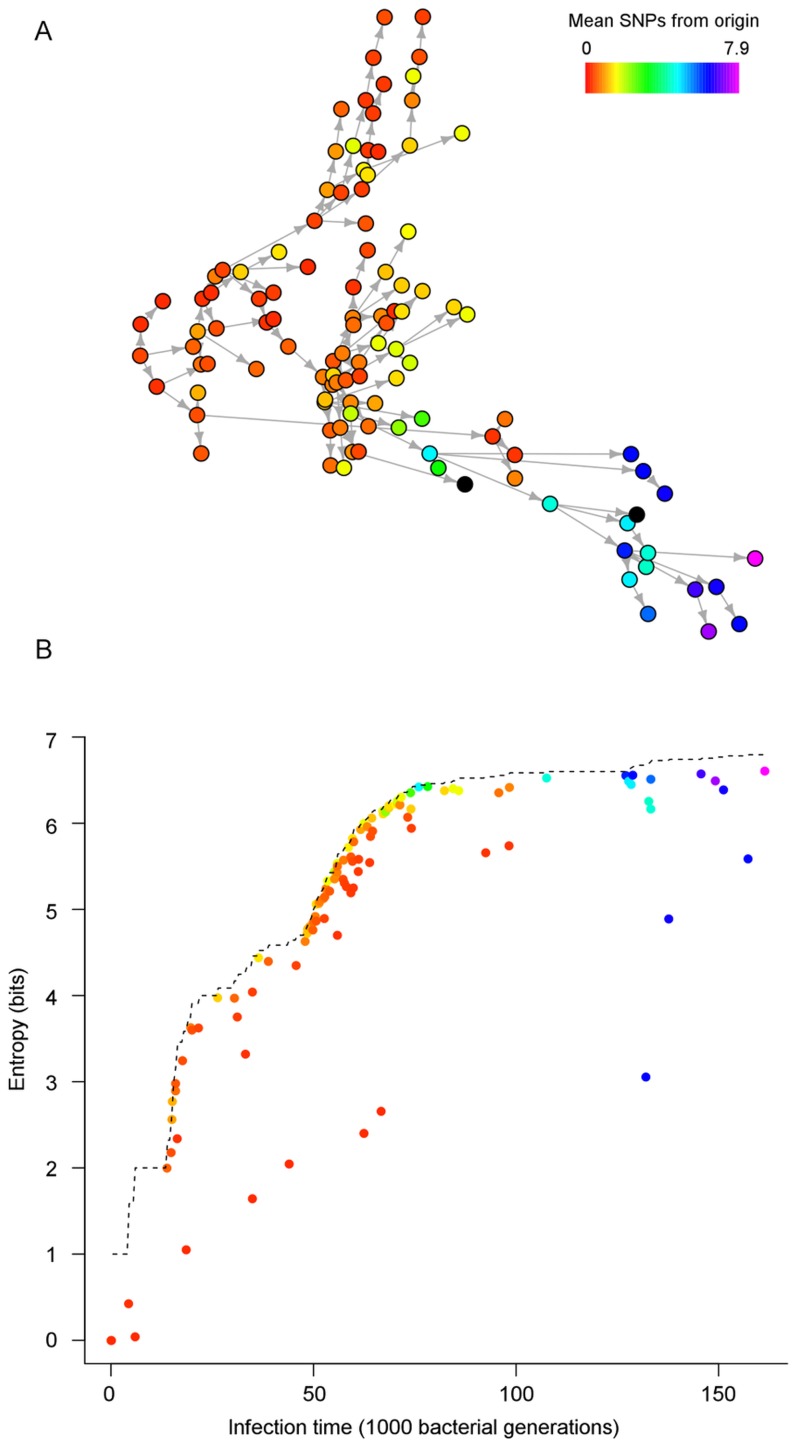
(A) Simulated SIR epidemic in a population with 250 initial susceptibles and one infective with an initially clonal infection. Samples were taken at random every 1000 generations from each individual. The color of the node indicates its mean genetic distance to the origin, based on observed sequences. Black nodes indicate individuals whose infectious period did not coincide with a sampling time. (**B**) The entropy for each node in an estimated transmission network, plotted against time of infection. The dashed line indicates the ‘no information’ case, in which sources are guessed at random (based on the order of infection only). Data were simulated with a mutation rate 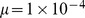, transmission rate 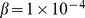 and removal rate 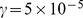.

We simulated epidemics under a variety of scenarios and found that generally for larger outbreaks, such that several infective individuals were present at any one time, the power to determine the routes of transmission was low. We supposed that we did not know the infection or removal times, only observing the correct order of infection. Table S1 gives area under the ROC curve values for estimated networks based on a selection of simulated datasets. In many cases, particularly for higher rates of infection and removal, we found that the ROC curve indicated no improvement on guessing transmission sources at random. However, we saw that distinct groups of individuals, representing large branches of the transmission network, may be distinguished from one another, indicating that gross features of the transmission network may be determined. Figure 4A shows a simulated epidemic in which nodes are colored according to their observed mean distance from the origin. Clearly later infections can be discriminated from cases further in the past, but a great deal of uncertainty exists among contemporary cases. Network reconstruction was more successful in scenarios where higher diversity could be established between host and recipient. As such, network reconstruction improved for long carriage times, low transmission rates, and high mutation rates (Table S1).

Network entropy may be used to evaluate the uncertainty arising under the network reconstruction approach (see *Materials and Methods*). As the outbreak progresses, the entropy of most nodes increases and is only modestly lower than that obtained from assigning an even probability to all preceding cases (Figure 4B). However, certain nodes are markedly less uncertain than the surrounding ones, indicating that for them, incorporating genetic distance considerably reduces the uncertainty of who infected them. In this outbreak, for example, the entropy distribution is bimodal, with 99 of the 112 nodes having entropy within one bit of random guessing.

**Figure 5:**
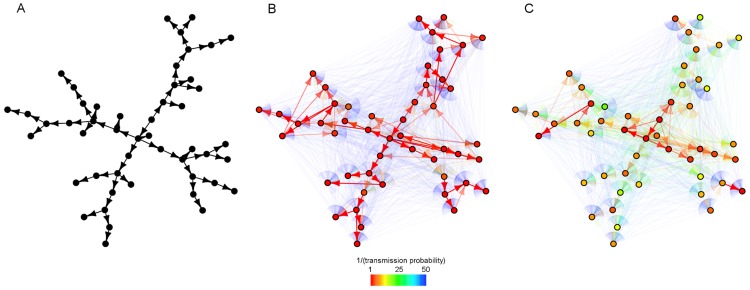
A simulated infection network (A) is estimated using importance factors of 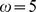 (B) and 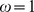 (C). The color of edges represents the probability of their existence, while the color of each node represents the highest probability assigned to any its potential sources (thus red indicating near-certainty about the source of a node). Data were simulated with a mutation rate 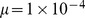, transmission rate 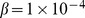 and removal rate 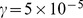.

In Figure 4, the infector of each node was identified with probability proportional to the inverse of the genetic distance between the populations, guaranteeing that some positive probability is assigned to the true infector. Entropy may be reduced (possibly at the expense of lowering the estimated probability of infection by the true infector) by increasing the relative probability of infection from nodes that are genetically close. Importance of similar nodes can be increased up to the point at which the closest node is selected with certainty, and the maximum directed spanning tree is selected, (equivalent to the *SeqTrack* method of network reconstruction [4]), resulting in zero entropy. Figure 5 shows the same network estimated with a varying importance factor. While some correct edges are estimated with a higher probability, several false connections are also estimated with little uncertainty. Precision is increased often at the expense of accuracy, and indeed increasing the importance factor for this network reduces the area under the ROC curve. Table S2 gives values for the area under the ROC curve for estimated networks under a particular simulated dataset, showing how accuracy declines as closer nodes are weighted more heavily. The true parent of a node has no guarantee of being the closest node, but is likely to belong to a group of genetically similar potential sources.

### Sampling

**Figure 6:**
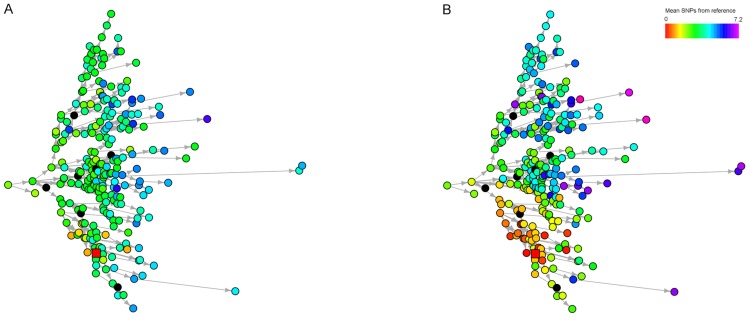
Simulated SIR epidemic in a population with 250 initial susceptibles and one infective with an initially clonal infection. A node was chosen at random during the epidemic (‘reference’, shown as a red square), and all other nodes are colored according to the observed mean genetic distance to the reference. (**A**) One isolate is sequenced from each individual. (**B**) Sampling occurs for all infectives every 100 generations. Data were simulated with a mutation rate 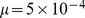, transmission rate 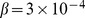 and removal rate 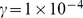.

Sampling strategies play an important role in the accuracy of the estimated network – while it is unsurprising that more frequent sampling results in reduced uncertainty, it is notable that even with perfect sampling, the uncertainty typically remains much too large to identify individual transmission routes. Figure 6 shows the same simulated outbreak, colored according to two different sampling strategies; firstly sequencing one isolate from each individual every 1000 bacterial generations, and secondly sequencing isolates ten times at each time point. In each plot, an arbitrarily chosen reference node is marked, to which each other node is compared. The second plot shows that the ‘neighborhood’, to which the reference node and its true source belong, may be discerned, genetically distinct from the rest of the outbreak. Increasing sampling frequency beyond this level does not considerably improve discrimination.

Selecting a single isolate per individual typically leads to a poor estimation of the transmission network. We found that the initial genotype often persisted throughout an epidemic, and remained the dominant genotype for a large number of infected individuals. Selecting a single isolate from each infective would result in a large number of individuals with an apparently genetically identical infection, providing little information about transmission. Multiple samples can reveal minor genotypes, which may be more informative. We found that in most reasonable settings, the reconstructed network based on single isolates was uncertain and inaccurate, sometimes worse than a random network.

## Discussion

Our work suggests that under a range of plausible scenarios considered here, it is not possible to determine transmission routes based upon sampled bacterial genetic distance data alone. For every infected individual in a large outbreak, there are several other individuals harboring a similar pathogen population who may be the true source of infection. Existing distance-based methods typically assume that a single isolate is obtained from each host, in which case the distance between hosts is simply the number of SNPs separating the two isolates. Sampling only one isolate per case can lead to poor estimates of genetic distance between individuals, and therefore inaccurate identification of transmission routes, often little better than assigning links at random. Increasing the sampling to obtain more than one sample per host may partially alleviate this problem; in this case, the genetic distance between two hosts may be estimated as the mean distance between isolates from one host and isolates from the other. The amount of sampling required depends on what one hopes to gain from the sequence data. Single isolates may be sufficient to rule out infection sources for individuals, based on large observed genetic distances. Repeated sampling may be used to identify clusters of infected individuals who host very similar bacterial populations, and therefore are likely to be close neighbors in the transmission network. This allows us to investigate more general trends in the progression of the outbreak, eg. spread between communities or countries, while individual events remain obscure.

A considerable degree of diversity is transmitted with even a small inoculum from the source, under the assumption that the inoculum is sampled randomly from the pathogen population infecting the source. We believe that this highlights the importance of establishing the degree of within-host diversity through multiple samples before attempting to infer transmission routes. Such sampling will also further our understanding of the transmission bottleneck for bacterial pathogens, as well as the effective population size.

Many of the parameters in our simulations are difficult to estimate for bacteria *in vivo*, and as such, few estimates exist. Moreover, population structure within a host may lead to divergence between the census and effective population sizes in each host [36]. To obtain results that would be widely applicable in spite of these uncertainties, we simulated transmission and carriage under a wide range of plausible parameter values for bacterial pathogens. Bottleneck size is a key factor in the onward transmission of diversity and network recovery – too small and resulting infections are homogeneous, too large and recipients share the same genotype distribution as the source.

In our inference of transmission routes, we have measured the average genetic distance between individuals across the span of the infectious period. If the removal rate is sufficiently low relative to the mutation rate, the genetic makeup of the pathogen population in an individual will vary considerably over time. As such, while a source and recipient may be genetically similar at the time of infection, the mean distance between observed samples may be higher. It may be possible to either restrict or weight the range of samples used in order to gauge the distribution of genotypes at a particular time; however, this comes at the expense of excluding potentially useful data. Using the mean genetic distance is not unreasonable if the length of carriage is small compared to the time required to accumulate significant diversity.

We have considered different sampling strategies, but have supposed that a large coverage of the infected population can be achieved. This may be reasonable for an outbreak in a small community, but inevitably, there may still be some missing links, especially when asymptomatic carriage could go undetected. Furthermore, we assumed that the order of infections is known. We have demonstrated that the reconstructed network accuracy is typically poor, even in the best-case scenario of near perfect observation.

We did not consider the possibility of repeated infectious contact, leading to infection from multiple sources. This could serve to increase the diversity within-host, further complicating the inference of transmission routes. In many settings, it is reasonable to assume that infectious individuals may come into contact with each other, and potentially transmit. In the case of vector-borne diseases, the vector (eg. a healthcare worker in nosocomial *S. aureus* transmission) may transiently carry multiple strains collected from one or more carriers, and pass this diversity on to recipients. If 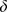 is the rate at which a novel SNP is introduced via reinfection, then the equilibrium level of diversity is increased to 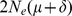. If the type(s) introduced upon reinfection are sufficiently dissimilar to the existing population, it may be possible to infer reinfection events. However, if the rate of infectious contact is high, most bacterial populations may contain artifacts from several disparate sources, preventing any kind of transmission analysis.

The ability to reconstruct transmission networks is dependent on both data and methodological limitations. While we cannot rule out the possibility of alternative methods using genetic distance data to provide superior network reconstructions, the framework we use here is flexible enough to investigate a range of relationships between genetic distance and transmission, under the widely used assumption that individuals hosting genetically similar pathogens are more likely to have been involved in a transmission event than those infected by more distantly related organisms.

In this study, we have made a number of assumptions. Firstly, we have used a discrete model of bacterial growth in which cells simultaneously divide and die at generational intervals. We have specified that a cell must divide or die at each generation, such that persisting without reproduction is not possible. Under this model, the effective population size is equal to the actual population size - incorporating cell survival without reproduction would only serve to reduce the effective population size, and therefore, the accumulation of diversity. Secondly, we have assumed neutral evolution; that is, there is no fitness advantage or cost associated with any mutation. Selection is likely to decrease the amount of instantaneous diversity within a population. The emergence of fitter mutations is likely to reduce the expected diversity, since fitter strains are more likely to tend towards fixation, eliminating weaker variants and their associated diversity. However, the effect of selective sweeps over time could increase the observed diversity in a longitudinal sample. Thirdly, we have assumed that an inoculum is composed of a random sample of bacteria from the entire colony. If the inoculum is not a random sample, the degree of diversity that is transmitted upon infectious contact may be much smaller. The suitability of this assumption may vary depending on the mode of transmission. However, we could consider the bottleneck size used here to represent the *effective* population size of the inoculum, rather than the true size. Finally, we have ignored the possibility of recombination. Further work would be required to explore the effect of each of these aspects in detail.

The observation of rare variants in cross-sectional samples from individual hosts may offer an alternative approach to identifying the transmission network. Each observation of a particular genotype must arise from a shared ancestor, assuming homoplasy is not possible. With perfect sampling, a genotype carried by only two individuals under these conditions indicates a transmission event between the pair. However, many isolates would need to be sequenced to detect such variation which is by definition rare. Such sampling is typically infeasible via standard genome sequencing, although deep sequencing may reveal uncommon SNPs, suggesting transmission between carriers. Metagenomic sampling may potentially be of great use in such an approach. Furthermore, such sampling may provide significant practical and financial advantages over collection and sequencing several individual samples. Future work may be conducted to investigate the performance of such an approach under a variety of scenarios, for viral as well as bacterial pathogens.

It may be possible to develop a genetic distance threshold such that any observed pair of isolates exceeding this value are deemed, to a given level of confidence, not to have arisen from directly linked cases. Such a threshold will depend on the bottleneck size, effective population size and mutation rate. As yet, no such limit has been justified theoretically, and appropriate data to investigate this are lacking.

This work highlights the need to better understand bacterial carriage and transmission at a cellular and molecular level. As yet, few studies have sequenced repeated samples from infected people, so the scale of within-host diversity is still unclear. Furthermore, key parameters such as effective population size and inoculum size are either highly uncertain or unknown for bacterial pathogens. If feasible, we recommend multiple isolates be sequenced per individual when collecting data to assess transmission routes.

While our work casts some doubt on the use of bacterial sequence data to identify individual transmission routes, there is certainly still much scope for its use in the analysis of disease transmission dynamics. Uncovering clusters of genetically similar isolates can be greatly informative for the spread of a disease between various subpopulations, such as households, schools and hospitals. By combining genomic data with additional information, such as estimated infection and removal times, contact patterns, social groups and geographic location, it may be possible to narrow the pool of potential sources down considerably. Genomic data and traditional ‘shoe-leather epidemiology’ methods may complement each other; each eliminating links that the other cannot rule out.

## Materials and Methods

### Bacterial simulation

Our simulation studies were based around a discrete-time bacterial fission model. We supposed that bacteria cells died at random with probability 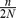, where 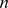 is the bacterial population in the previous generation, and 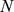 is the equilibrium population. The remaining cells divided, creating a mutant daughter cell with probability 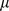, otherwise creating a genetically identical copy of the parent cell. Mutations introduced one nucleotide substitution at a random position in the genome, such that the genetic distance from parent to mutant was always one SNP. Neutral evolution is assumed. Under this model, the effective population size is equal to the size of the population; that is, 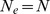 [37]. In the event of an infectious contact, an inoculum of size 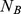 was separated from the original population, and allowed to grow and diversify independently. The inoculum was assumed to be a random sample from the original population.

In the epidemic simulations, we used a standard SIR model, in which each susceptible individual is exposed to an infection rate of 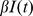 at time 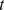, where 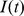 is the proportion of infected individuals at time 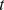. Infected individuals are then removed (through recovery or death) at a rate 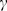. As we operated in a discrete-time framework, we used Poisson approximations to generate times of infection. For generation 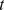, a given susceptible individual avoids infection with probability 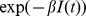. An individual infected in generation 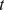 may transmit to another individual from generation 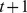 onwards. The source of a new infection is chosen uniformly at random from the pool of current infectives.

We assumed that the order of infection was known, and that all infective individuals were observed. Failure to identify routes of infection under these optimal conditions would provide little confidence that this could be achieved in a real world setting, where such information is rarely available.

### Identifying transmission routes

The relationships between isolates may be considered either directly from the sequence data, or from a matrix of observed genetic distances. The former category encompasses methods explicitly considering the evolutionary process, such as maximum likelihood and parsimony tree construction. Neighbor joining, UPGMA, minimum spanning tree construction and *SeqTrack* all belong to the latter. In this study, we were primarily interested in the relationship between individuals, rather than between bacterial specimens, and as such, did not adopt a phylogenetic approach. We instead weighted network edges according to the genetic distance matrix, supposing that the likelihood of direct infection having occurred was inversely related to the genetic distance.

Given the infective population is fully observed, a function may be defined to provide weight to each potential network edge. We assume this weight is inversely related to the genetic distance between the two nodes. This distance may be specified in various ways – here, we consider the mean genetic pairwise distance and the distance between the centroid of each group.

Let 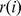 denote the set of sequences observed from individual 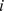, then the mean genetic distance between 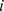 and 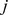 can be given as
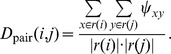
Alternatively, let 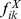 be the proportion of samples in 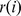 with a nucleotide 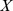 at locus 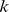. The distance between the centroids of 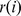 and 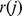 can be defined as
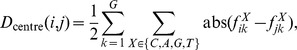
where 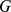 is the genome length, and 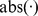 returns the absolute value. Unlike the pairwise distance, the centroid distance has the desirable property that 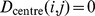 for all 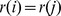; however, the converse is not true.

We calculate the relative probability that a particular transmission event occurred by considering the inverse of the chosen distance function 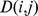;
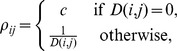
then we can define our weighting function as
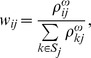
where 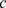 is a constant to determine the relative probability of a connection between individuals with identical genotype distributions, and 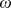 is a proximity factor by which the importance of close connections may be increased or decreased. The set 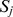 is the collection of potential sources that may infect person 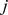. As 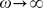, we consider the closest node to be the source with probability 1 (in the event of joint closest nodes, these are assigned equal probabilities). This then resembles a maximum weighted network. While we cannot rule out an alternative functional form providing an improved network reconstruction performance, the framework we use here is grounded in the assumption that genetically closer samples are more likely to be epidemiologically linked, and can represent a variety of relationships via specification of the parameters 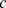 and 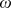. We used 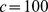 and 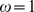 as baseline values, but explored variations of these values to investigate a range of relationships.

If the order of infection is known, then as 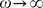, the resulting network is equivalent to the minimum directed spanning tree. Edmonds' algorithm finds a minimum directed spanning tree by identifying the lowest weighted incoming edge is selected for each node, before cycles in the graph are eliminated [38]. With a known order of infection, there will be no cycles in the graph, making the two approaches equivalent. This approach is implemented in *SeqTrack* [4].

Throughout our simulations, we have defined the genetic distance to be the number of SNPs between isolates. Equally, more complex distance metrics may be employed, for example, allowing transitions and transversions to have heterogeneous weighting (eg. [7]).

### Network accuracy and uncertainty

ROC curves indicate the accuracy of an estimated network, compared to the true network. A large area under the ROC curve indicates a good estimate of the true network [39].

We additionally considered the entropy of the estimated structure, as a measure of network uncertainty. Let 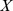 be a network with 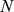 nodes. We define the entropy of a network node 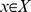 to be
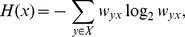
where 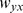 is the weight we assign to a directed edge existing from 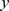 to 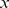, and 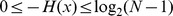. Highly negative entropy signifies uncertainty surrounding the identity of the parent of the node.

All simulations and analyses were performed using the statistical software R 3.0.1. Epidemic networks were plotted using the *igraph* package [40].

## Supporting Information

Figure S1 **The effect of population size and mutation rate on the accumulation of within-host diversity.** The propagation of diversity in an initially clonal population. For various mutation rates and population sizes, we simulated 100,000 generations of growth and recorded the expected pairwise distance. For each scenario, we repeated the simulation 50 times, plotting the mean diversity, and the 95% confidence interval.
(TIF)

Figure S2 **Diversity arising after a bottleneck.** The observed diversity of a population having passed through bottlenecks of various size. For each level, we simulated 1000 independent bottlenecks, and measured the Simpson's diversity index (green) and the expected pairwise distance (blue).
(TIF)

Figure S3 **Stochastic realizations of a series of bottlenecks on a diverse population.** Six simulations of a chain of bottleneck events. In each simulation, the initial population is specified as ten genotypes in equal frequency, with an expected pairwise distance of 5 SNPs. For each scenario, the upper graph depicts the changing genotype frequencies across bottlenecks, while the lower plot shows the expected pairwise distance.
(TIF)

Figure S4 **The reduction in host diversity caused by repeated bottlenecks.** The effect of repeated bottlenecks on a diverse population. For a given initial population of 10 genotypes, with an expected pairwise distance of 5 SNPs, we passed a population through a series of 25 bottlenecks of various sizes, allowing 1000 generations of regrowth and mutation after each event. For each scenario, we repeated the simulation 50 times, plotting the mean diversity and the 95% confidence interval.
(TIF)

Table S1
**Area under the ROC curve (AUC) for estimated transmission networks, based on simulated epidemics for a range of parameter values.** For all reconstructions, it is assumed that the order of infection is known, but infection and removal times are not known. Bold figures indicate values exceeding the AUC for an uninformed network, given the correct order of infection, which was approximately 0.66. The rate of removal was specified such that 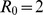.(DOC)

Table S2
**AUC for estimated transmission networks, using various values of proximity factor** 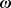. The epidemic considered here corresponds to that shown in figure 5.(DOC)

Text S1 **Demonstration that the expected sample diversity is equal to original diversity, regardless of the size of the bottleneck.**
(DOC)
