# Supplementary figures and images for: Within-Host Bacterial Diversity Hinders Accurate Reconstruction of Transmission Networks from Genomic Distance Data
Source: PLoS Comput Biol. 2014 Mar 27;10(3):e1003549. doi: 10.1371/journal.pcbi.1003549 (PMC3967931; doi:10.1371/journal.pcbi.1003549)

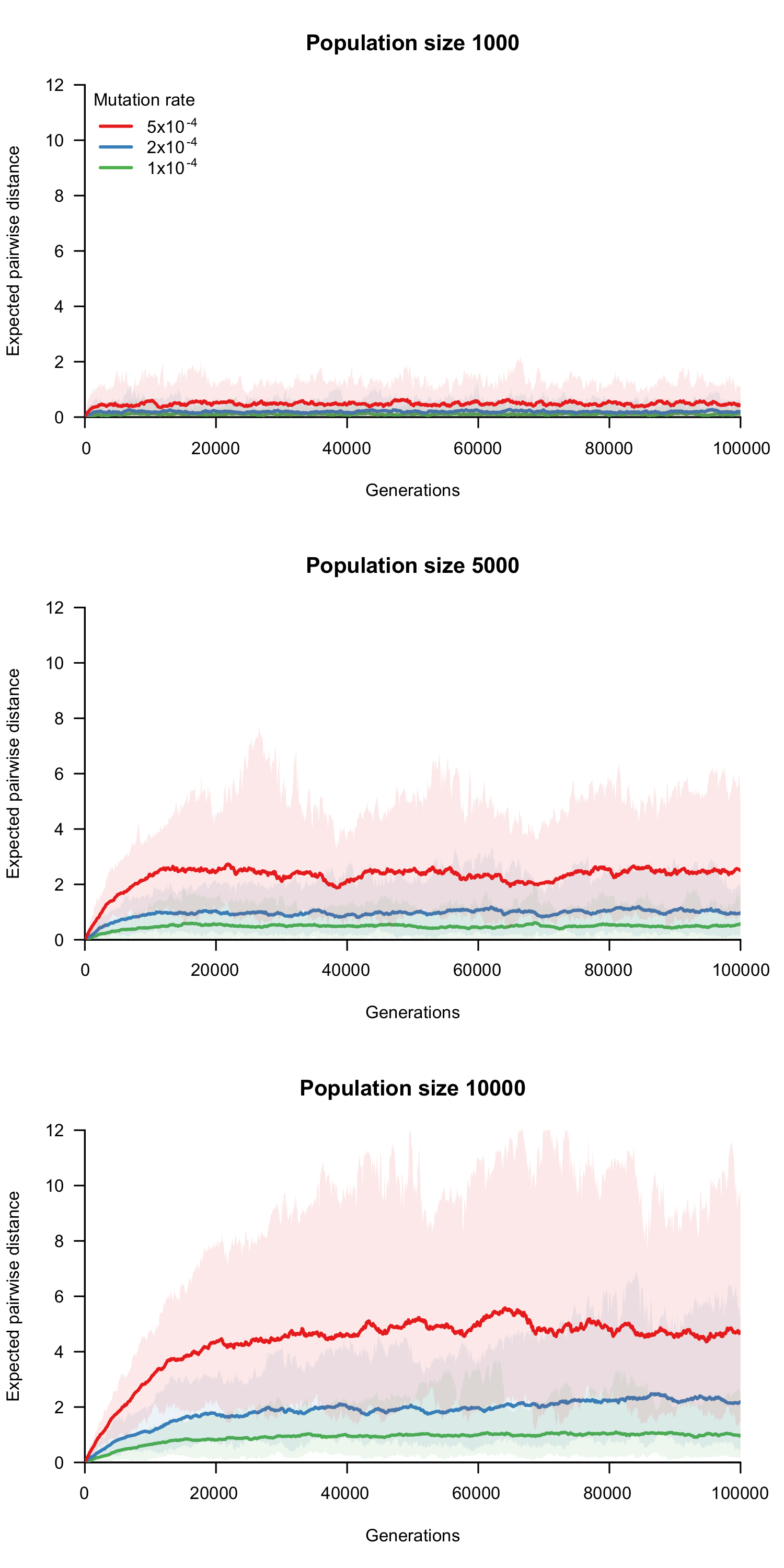

Supplement: Figure S1 — The effect of population size and mutation rate on the accumulation of within-host diversity. The propagation of diversity in an initially clonal population. For various mutation rates and population sizes, we simulated 100,000 generations of growth and recorded the expected pairwise distance. For each scenario, we repeated the simulation 50 times, plotting the mean diversity, and the 95% confidence interval. (TIF) [file pcbi.1003549.s001.tif]

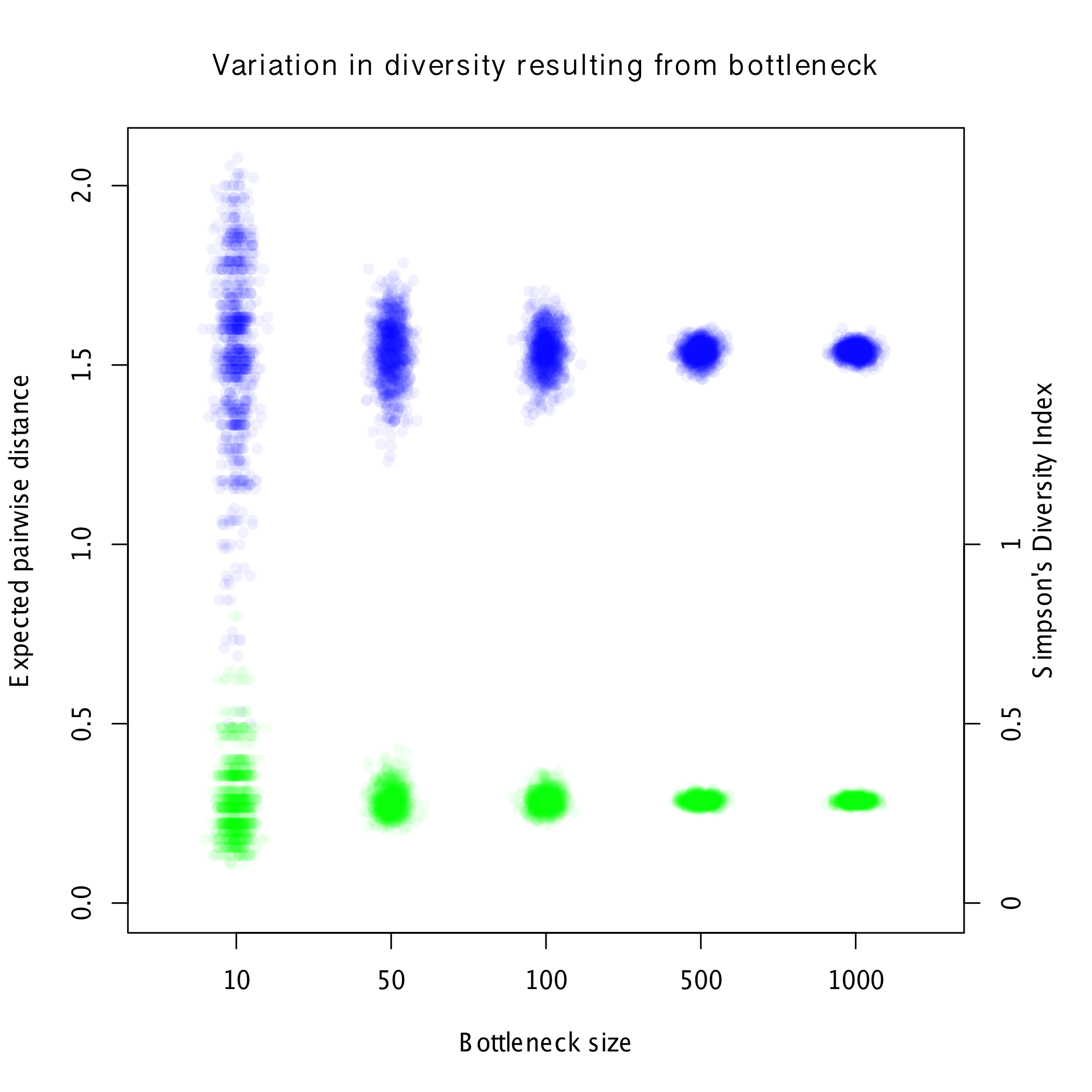

Supplement: Figure S2 — Diversity arising after a bottleneck. The observed diversity of a population having passed through bottlenecks of various size. For each level, we simulated 1000 independent bottlenecks, and measured the Simpson's diversity index (green) and the expected pairwise distance (blue). (TIF) [file pcbi.1003549.s002.tif]

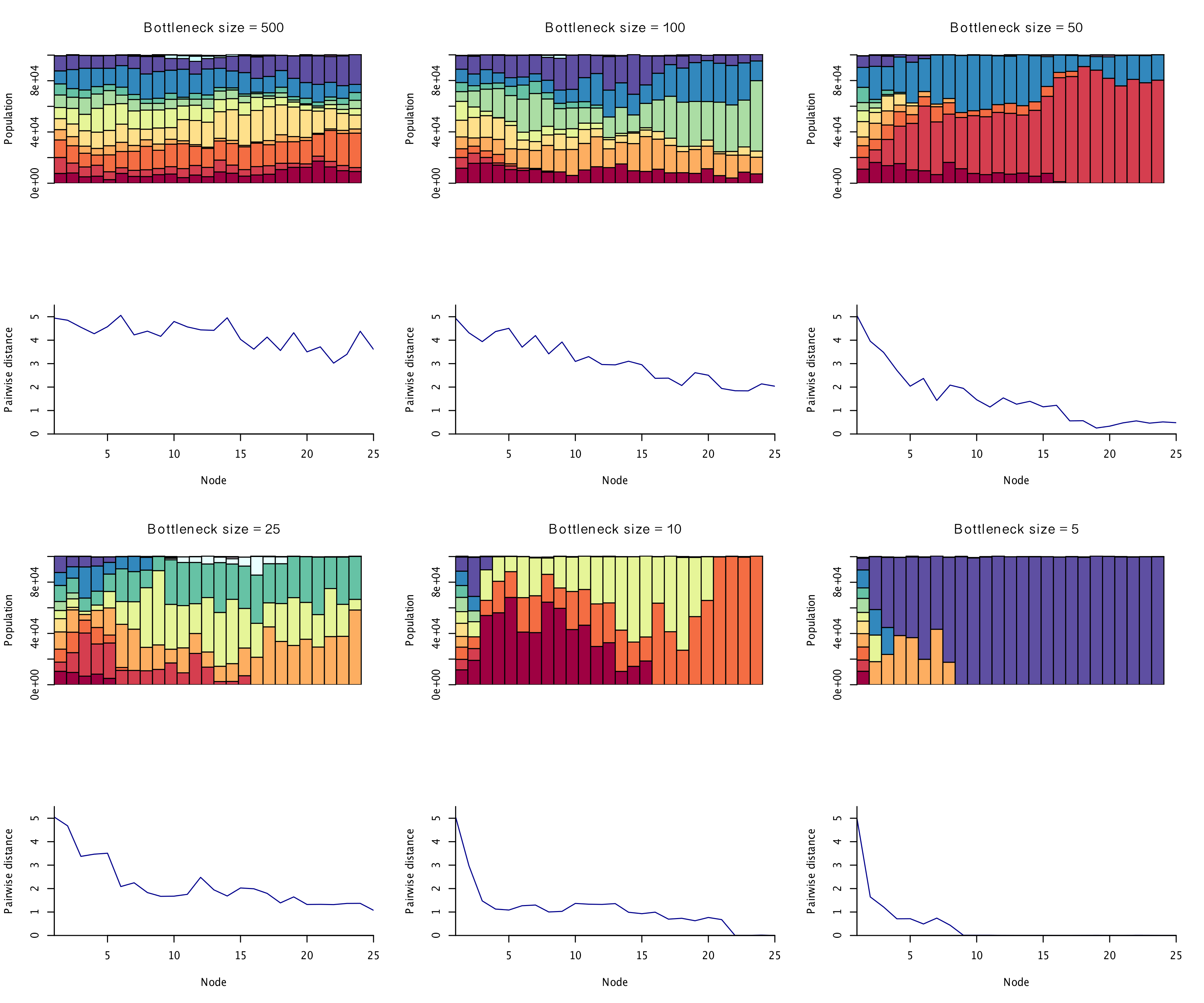

Supplement: Figure S3 — Stochastic realizations of a series of bottlenecks on a diverse population. Six simulations of a chain of bottleneck events. In each simulation, the initial population is specified as ten genotypes in equal frequency, with an expected pairwise distance of 5 SNPs. For each scenario, the upper graph depicts the changing genotype frequencies across bottlenecks, while the lower plot shows the expected pairwise distance. (TIF) [file pcbi.1003549.s003.tif]

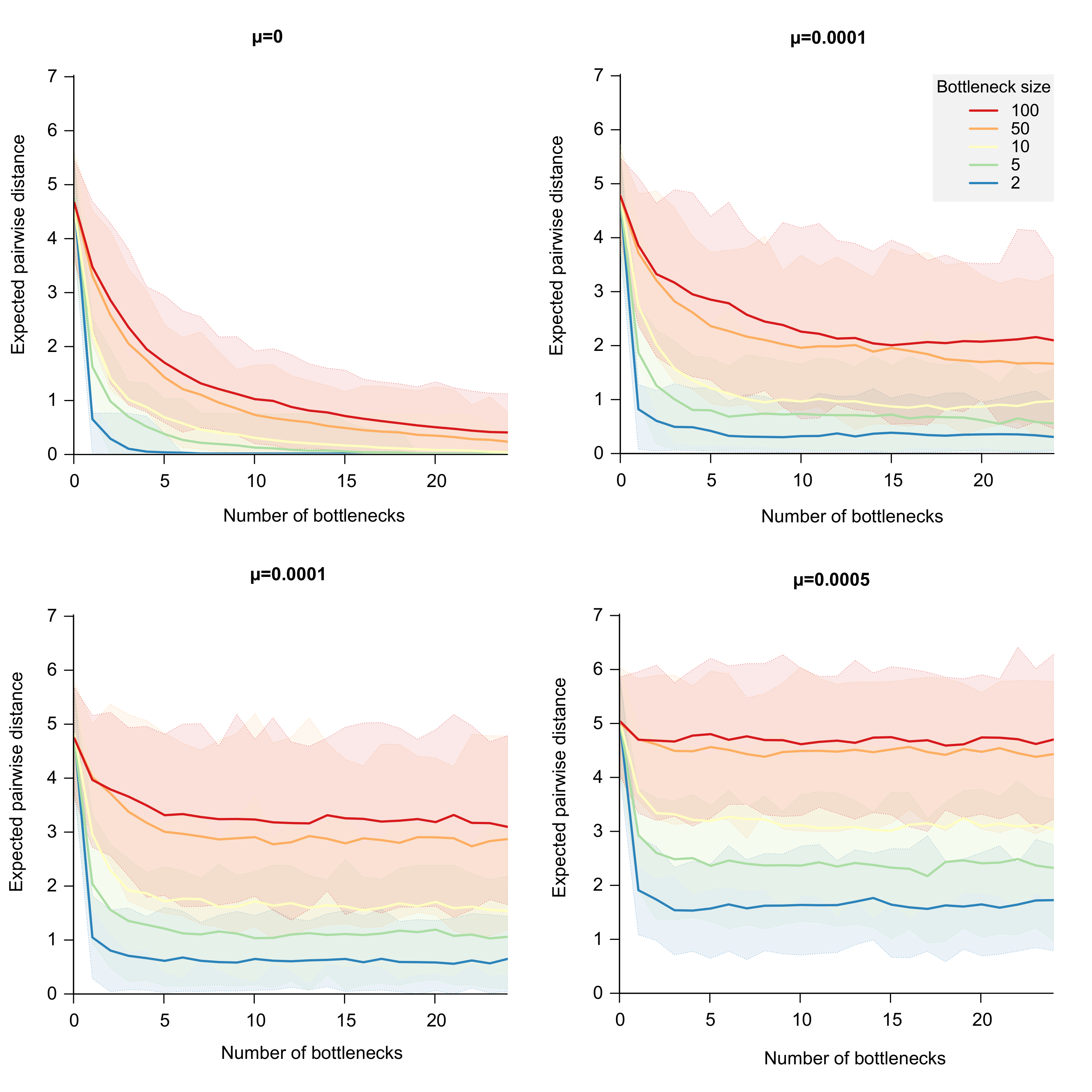

Supplement: Figure S4 — The reduction in host diversity caused by repeated bottlenecks. The effect of repeated bottlenecks on a diverse population. For a given initial population of 10 genotypes, with an expected pairwise distance of 5 SNPs, we passed a population through a series of 25 bottlenecks of various sizes, allowing 1000 generations of regrowth and mutation after each event. For each scenario, we repeated the simulation 50 times, plotting the mean diversity and the 95% confidence interval. (TIF) [file pcbi.1003549.s004.tif]
