# Supplementary material for: Within-Host Bacterial Diversity Hinders Accurate Reconstruction of Transmission Networks from Genomic Distance Data
Source: PLoS Comput Biol. 2014 Mar 27;10(3):e1003549. doi: 10.1371/journal.pcbi.1003549 (PMC3967931; doi:10.1371/journal.pcbi.1003549)
Supplement: Table S1 — Area under the ROC curve (AUC) for estimated transmission networks, based on simulated epidemics for a range of parameter values. For all reconstructions, it is assumed that the order of infection is known, but infection and removal times are not known. Bold figures indicate values exceeding the AUC for an uninformed network, given the correct order of infection, which was approximately 0.66. The rate of removal was specified such that . (DOC) [file pcbi.1003549.s005.doc]

|  |  | Bottleneck size | | | |
| --- | --- | --- | --- | --- | --- |
| Transmission rate (x10^-4^) | Mutation rate (x10^-4^) | 1 | 5 | 10 | 25 |
| 1 | 1 | **0.85** | **0.84** | **0.70** | **0.66** |
|  | 2 | **0.79** | **0.84** | **0.73** | **0.81** |
|  | 5 | **0.85** | **0.88** | **0.80** | **0.79** |
|  | 10 | **0.79** | **0.90** | **0.82** | **0.81** |
| 5 | 1 | **0.72** | 0.64 | 0.64 | **0.71** |
|  | 2 | **0.73** | **0.67** | 0.61 | **0.70** |
|  | 5 | **0.73** | **0.75** | **0.72** | **0.79** |
|  | 10 | **0.80** | **0.70** | **0.75** | **0.77** |
| 10 | 1 | **0.67** | **0.70** | 0.65 | 0.64 |
|  | 2 | **0.75** | 0.65 | 0.63 | 0.62 |
|  | 5 | 0.62 | 0.59 | 0.64 | 0.60 |
|  | 10 | **0.78** | 0.59 | 0.64 | 0.59 |
