# Supplementary material for: Within-Host Bacterial Diversity Hinders Accurate Reconstruction of Transmission Networks from Genomic Distance Data
Source: PLoS Comput Biol. 2014 Mar 27;10(3):e1003549. doi: 10.1371/journal.pcbi.1003549 (PMC3967931; doi:10.1371/journal.pcbi.1003549)
Supplement: Table S2 — AUC for estimated transmission networks, using various values of proximity factor . The epidemic considered here corresponds to that shown in figure 5. (DOC) [file pcbi.1003549.s006.doc]

| Proximity factor, $\omega$ | AUC |
| --- | --- |
| 1 | 0.917 |
| 2 | 0.914 |
| 5 | 0.901 |
| 10 | 0.898 |
