# Supplementary material for: Within-Host Bacterial Diversity Hinders Accurate Reconstruction of Transmission Networks from Genomic Distance Data
Source: PLoS Comput Biol. 2014 Mar 27;10(3):e1003549. doi: 10.1371/journal.pcbi.1003549 (PMC3967931; doi:10.1371/journal.pcbi.1003549)
Supplement: Text S1 — Demonstration that the expected sample diversity is equal to original diversity, regardless of the size of the bottleneck. (DOC) [file pcbi.1003549.s007.doc]

Supporting Information

*Proof that the expected sample diversity is equal to original diversity, regardless of the size of the bottleneck*

Consider a large population composed of $k$ genotypes, in proportions $\pi_{1},...,\pi_{k}$. The diversity of this population can be measured by Simpson’s diversity index $D_{s}$, or the expected pairwise genetic distance, . We take a random sample of size $N_{B}$, observing $\hat{n}_{1},...,\hat{n}_{k}$ of each genotype (or equivalently, proportions$\hat{\pi}_{1},...,\hat{\pi}_{k}$), and thus measure the sample diversity of the new population as$\widehat{D_{s}}$, or. Using the properties of the multinomial distribution, we have

$E(\hat{\pi}_{i})=\pi_{i}$ ,

and

$\text{Var}(\hat{\pi}_{i})=\frac{\text{Var}(\hat{n}_{i})}{N_{B}^{2}}=\frac{\pi_{i}(1-\pi_{i})}{N_{B}}$ ,

so therefore

$$\begin{matrix} E(\widehat{D_{s}})=E\left(\sum_{i=1}^{k} \frac{\hat{n}_{i}(\hat{n}_{i}-1)}{N_{B}(N_{B}-1)}\right)=\frac{1}{N_{B}(N_{B}-1)}\sum_{i=1}^{k} \left[E(\hat{n}_{i}^{2})-E(\hat{n}_{i})\right] \\ =\frac{1}{N_{B}(N_{B}-1)}\sum_{i=1}^{k} \left[\text{Var}(\hat{n}_{i})+E(\hat{n}_{i})^{2}-N_{B}\pi_{i}\right] \\ =\frac{1}{N_{B}(N_{B}-1)}\sum_{i=1}^{k} \left[N_{B}\pi_{i}(1-\pi_{i})+N_{B}^{2}\pi_{i}^{2}-N_{B}\pi_{i}\right]=\sum_{i=1}^{k} \pi_{i}^{2}=D_{s} \end{matrix}$$

and
